# Introducing Open Highlights: Highlighting Open Access Research from PLOS and Beyond

**DOI:** 10.1371/journal.pbio.1002514

**Published:** 2016-07-11

**Authors:** The PLOS Biology Editors

**Affiliations:** The *PLOS Biology* editors are: Ines Alvarez-Garcia, Christine Ferguson, Emma Ganley, Gabriel Gasque, Liza Gross, Holly Ironfield, Christina Kary, Lauren Richardson, Roland Roberts and Hashi Wijayatilake

## Abstract

PLOS Biology announces a new article type, Open Highlights, which uses a recent research article to nucleate a short synthesis of up to ten related research articles from other PLOS journals and from the wider Open Access corpus.

When *PLOS Biology* was launched back in 2003, part of our remit was to broaden access to scientific research, not only in the literal sense via our open access publishing model, but also figuratively by offering a level of unpackaging and contextualisation of scientific results. Initially, we achieved this with our Synopses, which were penned by staff and freelance writers and focussed on individual research articles.

In recent times we’ve we spotted a missed opportunity; PLOS publishes an amazing scope of research, with fantastic papers appearing in all seven journals. As part of our contribution to helping readers grapple with this large body of science, we’ve decided to broaden our purview and highlight work from across all PLOS journals alongside individual articles published by *PLOS Biology*. Replacing Synopses with a format we’ve called “Open Highlights” allows us editors to provide more context for the findings published in our journal, to link it to related work of interest, to help curate PLOS content, and to highlight the bigger picture.

We’ve published 965 Synopses over the last 13 years; from the first [1] to the last [2], each has been read by thousands of people, and we hope that they’ve helped to widen the appeal and enhance the accessibility of the research we’ve published. With our new Open Highlights format, we hope to achieve more of the same, and casting an even wider net will allow us to draw together multiple strands of research around a theme. In addition, the format affords us the luxury of being able to highlight Open Access research from non-PLOS journals too.

We successfully trialled this idea on the PLOS Biologue blog platform with a series of posts called “Slices of PLOS,” using recent *PLOS Biology* research articles as keystones around which to nucleate a short synthesis of up to ten related research articles from other PLOS journals and from the wider Open Access corpus. Covering broad themes such as tomatoes [3] and bacterial quorum-sensing [4], each blog post is accompanied by a Collection [5] of the papers explored, allowing readers tantalised by the “Slice” to easily browse the research articles themselves. These Slices of PLOS have been well received by our community and we’re delighted to be extending them into *PLOS Biology* itself (Fig 1); we see this as a service for the authors of Open Access articles and also as a means to facilitate scientific engagement with our curious public.

**Fig 1:**
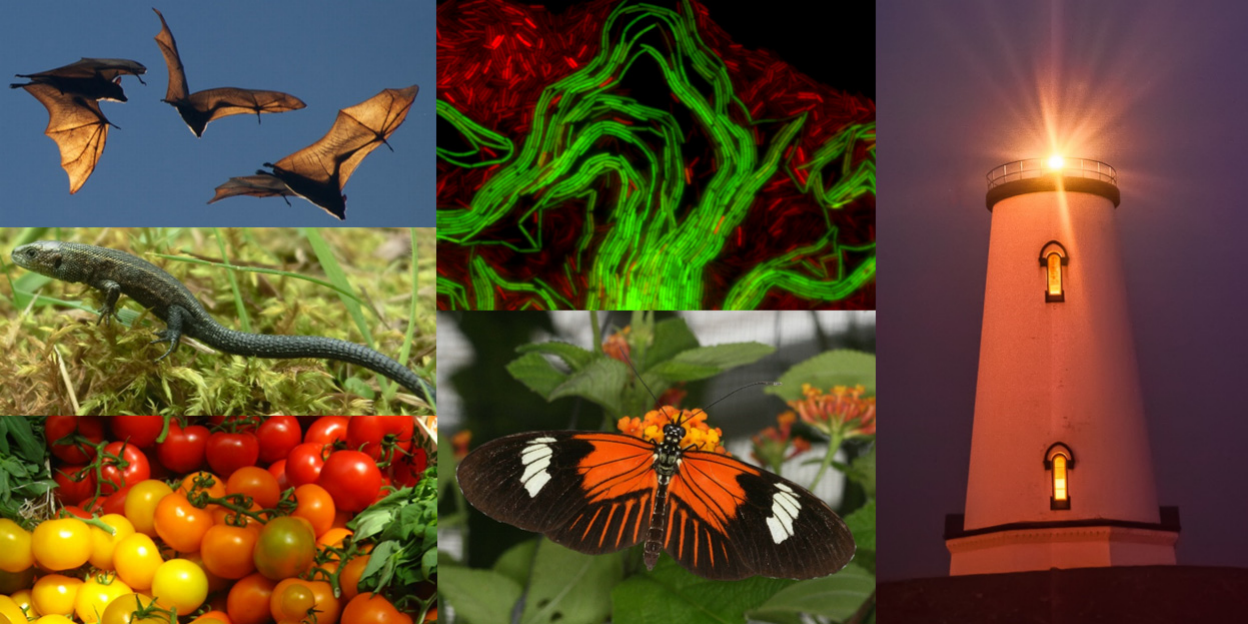
“Slices of PLOS” Become “Open Highlights.” The successful format of our PLOS Biologue series of “Slices of PLOS” (left) will now become the new journal article type Open Highlights (right). *Image credits (clockwise from top left)*: *Flickr user pldms*, *Jordi van Gestel*, *Flickr user puliarfanita*, *Chris Jiggins*, *Flickr user adactio*, *Elvire Bestion*.

By transforming this blog format into a journal article, we can use *PLOS Biology*’s higher visibility to bring the “Slice” approach to a wider readership and thereby enhance the discoverability of the Open Access research discussed. Thus Slice of PLOS has grown wings to become Open Highlights, and the first one is published today [6]. To find out more about Slices of PLOS and Open Highlights, read the related blog post [7] and drop in on the Open Highlights Collection page [8]. We hope you’ll enjoy this new format and that our Open Highlights will lead you to papers that you might not otherwise have encountered.
